# Application of video feedback in assessment skills training with autism level 1 screening

**DOI:** 10.3389/fpsyt.2025.1658102

**Published:** 2026-02-24

**Authors:** Xiang Long, Siming Dong, Yidong Zhu, Qiong Xu, Xia Gao, Haihui Wu, Pingping Xu, Jiang Hong, Hao Chen, Xiu Xu

**Affiliations:** 1 Department of Child Health, Jiading Maternity and Infant Health Hospital, Shanghai, China; 2 Department of Preventive Medicine and Health Education School of Public Health, Fudan University, Shanghai, China; 3 Department of Child Health, Children’s Hospital of Fudan University, Shanghai, China; 4 Department of Maternal and Child Health, Fudan University, Shanghai, China

**Keywords:** autism spectrum disorder, early symptom, screening technology, short video, teaching application

## Abstract

**Objective:**

To apply video feedback method in the training of primary screening and assessment skills for autism among community child care workers, and evaluate its effectiveness, providing ideas for future operational training at the grassroots level.

**Methods:**

After conducting centralized training and supervision on the first level screening of autism for community child care workers, they will shoot videos of the first level screening operation skills according to the key points. They will use monthly community meetings to report on the videos and discuss and analyze them. After a period of time, they will provide feedback on the videos again to enhance the first level screening skills of community child care workers for autism. Evaluate the effectiveness of video feedback method in learning primary screening skills for autism through video ratings, questionnaire surveys, interviews, and other methods.

**Results:**

After three consecutive months of video feedback training, 58 grassroots staff members in the district mastered the key operational points of autism screening through behavioral observation assessments. This standardized the first-level autism screening process across 13 community health service centers and 18 pediatric care clinics in Jiading District. Participants believe that the video feedback method has a good effect on skill learning. After conducting video feedback learning, the child protection team’s performance in several indicators such as reasonable positioning during screening (*P* = 0.022), parental interference (*P* = 0.029), guidance language (*P* = 0.002), and body movements in language items (*P* = 0.016) has improved, and the overall video score has also increased (*P* < 0.001).

**Conclusion:**

After the centralized operational training, multiple video feedbacks may effectively improve the screening.

## 1 Introduction

Autism spectrum disorder (Autism Spectrum Disorder, ASD) is a neurodevelopmental disorder characterized primarily by impairments in social interaction, deficits in verbal and nonverbal communication, and restricted interests and repetitive behaviors (1). The disorder typically has an onset before the age of 3 years. Early diagnosis and long-term, systematic intervention can maximize improvements in prognosis (2). As a pilot district of the Shanghai Child Mental Health Program, Jiading District began implementing an early ASD screening–intervention initiative in June 2021. In accordance with program requirements, the screening–intervention framework comprises (1): primary screening: community health service centers provide ASD screening for children aged 18–24 months (2); secondary screening: children with abnormal results are referred to the district maternal and child health hospital for further screening and monitoring; and (3) tertiary assessment: those with persistent abnormalities are further referred to the Children’s Hospital of Fudan University for diagnostic evaluation. At program initiation, experts from the Children’s Hospital of Fudan University conducted on-site training in Jiading, delivering instruction on the primary screening workflow and procedures. One month later, these experts were divided into three teams to supervise the quality of primary screening assessments conducted by frontline child health staff across the 13 community health service centers. The supervision found that only one center met the expert group’s standard, whereas the remaining 12 centers required rapid improvement in primary screening performance to ensure that child health outpatient staff across the district could implement standardized screening procedures. Accordingly, the Child Health Department of the Jiading District maternal and child health hospital summarized the assessment workflow and key operational points for the two behavioral observations in the primary screening and organized community child health staff to learn these procedures. Video-feedback sessions were conducted during the monthly child health routine meetings. After three rounds of video-feedback exchange, primary screening activities in all 13 community health service centers met the program requirements. To date, the screen-positive rate for primary ASD screening across the 13 community health service centers in Jiading District has gradually increased from 0.86% at program initiation to 2.43%. Although this rate increased with program implementation, it represents a process indicator and should be interpreted in conjunction with subsequent referral and diagnostic outcomes to evaluate its implications for screening accuracy.

In addition, growing evidence indicates that early identification and early intervention for ASD can substantially improve long-term outcomes. However, in real-world primary care settings, screening implementation often faces challenges: while checklist items may be fixed, behavioral observation is more subjective and operational consistency can be insufficient, leading to unstable screening quality. To strengthen frontline providers’ ability to identify high-risk behavioral cues and to standardize procedures during routine well-child visits, a training and supervision approach is needed that is repeatable, shareable at the team level, and amenable to quality control. Video feedback can capture key operational details in real clinical contexts; repeated review and structured discussion can reinforce critical points, thereby promoting procedural standardization and skill consolidation.

## 2 Objects and methods

### 2.1 Subjects

This project was implemented in the child health outpatient clinics of the 13 community health service centers in Jiading District and in the district maternal and child health institution. During the start-up phase, centralized training (on-site weekend sessions) was provided to personnel in child health–related positions across the district. In the subsequent video-feedback phase, primary screening procedure videos were recorded and presented on a team basis. A total of 58 frontline staff members (including full-time and part-time personnel) participated in the video-feedback activities for three consecutive months and submitted videos of primary screening procedures. Child health services in Jiading District comprise the child health department of the district maternal and child health institution and the child health departments of 13 community health service centers. In addition to providing high-risk child outpatient services, the district maternal and child health institution is responsible for professional guidance and supervision of child health services in the 13 community centers. Across the district, the 13 community health service centers operate 18 child health outpatient clinics, serving approximately 21,100 children aged 0–3 years. The total child health workforce comprises more than 85 staff members, including 51 full-time and 28 part-time personnel; 31 are licensed physicians (including 6 licensed pediatricians).

The study included 58 frontline staff members who participated in the three-month video-feedback phase and whose submitted primary screening procedure videos all passed expert review by specialists from the Children’s Hospital of Fudan University, including 51 full-time and 7 part-time staff members.

### 2.2 Methods

#### 2.2.1 Training content

Primary ASD screening (CHAT-A). The CHAT-A is a parent-completed screening questionnaire used in this project. It comprises 23 fixed items with standardized instructions, which facilitates implementation in primary care settings. Its item framework is based on M-CHAT–related screening constructs and is used for parent-reported primary screening of children aged 18–24 months (3) (4).

Assessment of two social high-risk warning behaviors (Binomial Observation Test, BOT). The BOT includes two behavioral observations (1): response to name calling; and (2) response to verbal instructions (following commands). During routine well-child visits in a naturalistic setting, both the physician and the caregiver independently initiate name calling and simple verbal instructions, while observing whether the child shows responsive behaviors such as turning toward the caller, making eye contact, vocalizing, or carrying out the instruction. BOT is considered positive if any observation indicates insufficient or absent responsiveness.

**Figure 1:**
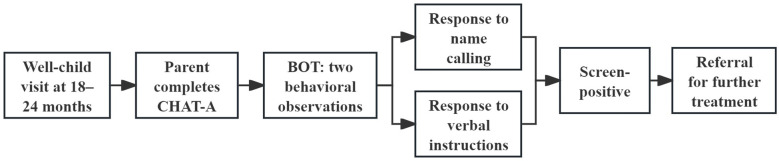
Two-step primary ASD screening workflow and referral pathway (CHAT-A + BOT).

Integrated determination of primary screening results. Primary screening results are determined by combining the two components. A child is classified as screen-positive at the primary level if either the parent questionnaire (CHAT-A) or any BOT component is positive, and the child then enters the subsequent referral pathway. Among these components, the physician-led behavioral observation is a critical element for identifying high-risk children at the primary screening stage and is therefore a key focus of subsequent screening supervision (**Figure 1**).

#### 2.2.2 Supervision quality control

In July 2021 (one month after the centralized training), the Children’s Hospital of Fudan University served as the expert technical support institution and was responsible for training and supervisory guidance, whereas the district maternal and child health hospital acted as the district-level management and quality-control body, overseeing organization, implementation, and routine supervision. A joint supervisory team was formed and divided into three supervisory groups to conduct on-site quality-control supervision across community child health outpatient clinics throughout the district. Supervision indicated that implementation of the parent-completed questionnaire (CHAT-A) was relatively consistent at the primary level, owing to its fixed items and standardized instructions. In contrast, the main challenges were concentrated in the BOT component. As BOT relies on behavioral observation, operational quality was susceptible to multiple on-site factors, leading to variability in implementation. These interfering factors primarily included the screening environment and equipment configuration, table height and seating arrangement, preparation of materials, pre-assessment communication with parents, and operational precautions during the assessment process. Following supervision, the district maternal and child health hospital developed a “screening precautions and key points checklist” based on expert recommendations and disseminated it to the 13 community centers to support subsequent quality improvement.

#### 2.2.3 Video feedback

Video recording. From August to October 2021, each community health service center organized video recording and presentation at the team level. After implementing improvements based on key points identified in primary screening, teams recorded videos of routine primary ASD screening procedures during daily practice. Each team reviewed and selected two to three primary screening videos that reflected its improvement focus for that month, which were then submitted for discussion at the subsequent month’s video-feedback meeting.

Video review and discussion. Video review was integrated into the monthly district-level child health routine meetings. Each community health service center took turns presenting the procedural changes implemented during that month’s video recording and discussing factors that could further improve screening performance. The remaining 12 community centers and the district maternal and child health hospital reviewed the submitted videos and conducted a structured appraisal of key points. Discussions focused on technical/professional elements, organizational factors, and the effectiveness of work processes. Teams also shared practical experience regarding screening procedures and communication with parents.

#### 2.2.4 Outcome measures and video scoring procedure

Two physicians from the project team at the district maternal and child health hospital served as evaluators. They reviewed the participant videos presented at each video-feedback meeting and assessed screening performance and operational execution, including: examiner position, equipment preparation, screening environment, management of parental interference, standardized verbal instructions, initiation timing, waiting time, number of operations, use of physical gestures during language items, and operational sequence. These 10 items were treated as key performance indicators and scored dichotomously (1 point if the criterion was met; 0 otherwise), yielding a total score ranging from 0 to 10 (5).

Specifically (1): Examiner position was defined as maintaining an appropriate distance and eye-level alignment with the child to ensure clear delivery of name-calling and instructions, while avoiding positioning that distracts the child or obstructs observation (2); Equipment preparation referred to having necessary materials, toys, and recording/registration tools prepared and placed for efficient use (3); Screening environment referred to a relatively quiet setting with distractions controlled to meet basic conditions for observing child responses (4); Parental interference management referred to communicating with parents in advance and minimizing prompts, answering on behalf of the child, or physical guidance during observation (5); Standardized verbal instructions referred to clear and concise name-calling and commands consistent with the project protocol, avoiding repeated explanations or leading cues (6); Initiation timing referred to initiating name-calling/commands when the child’s attention was relatively stable, rather than during crying or high-interference states (7); Waiting time referred to providing an adequate response window before proceeding to the next step, avoiding overly rapid progression (8); Number of operations referred to completing the prescribed number of name-calling/commands as specified, without omission or arbitrary increase/decrease (9); Physical gestures during language items referred to avoiding additional gestures or pointing that might cue the child when delivering verbal instructions; and (10) Operational sequence referred to completing the two behavioral observation steps in the order specified by the protocol.

The two evaluators scored independently. In cases of disagreement, the evaluators jointly reviewed the video and reached consensus; the final consensus score was used as the video score.

### 2.3 Survey questionnaire

The questionnaire was administered once after completion of the three-month video-feedback program and was collected within the specified period. Survey content included: (1) demographic and professional characteristics (age, sex, educational level, affiliated community health service center, department, professional title, years of practice, and duration of involvement in primary ASD screening); and (2) video-feedback–related information (team size, team roles, frequency and duration of video recording, self-rated video quality, perceived quality of other teams’ videos, and perceived effectiveness and advantages of video feedback). Questionnaires were deemed invalid and excluded from statistical analyses if they contained missing key items, logical inconsistencies, or large amounts of repetitive/meaningless responses.

### 2.4 Qualitative interviews

After completion of the video-feedback program, a qualitative approach was adopted, and seven staff members from different community health service centers were recruited for a focus group discussion. Interview topics included: (1) perceptions of ASD screening training among community child health staff; (2) experiences with implementing video feedback; (3) perceived improvement in operational skills attributable to video feedback; (4) concerns regarding the safety and privacy of video recording; and (5) perceived differences between video feedback and conventional on-site supervision. Data were collected using a semi-structured interview approach. Qualitative data collection and the final sample size were determined according to the principle of information saturation.

### 2.5 Data analysis

Data were analyzed using SPSS version 26.0. The team served as the unit of analysis. Paired comparisons were performed between the first and second video submissions for each team. Outcomes included item-level scores for the 10 key checklist items (scored 0 or 1) and the total score (range 0–10). Paired comparisons were conducted using paired t-tests, with a two-sided P value < 0.05 considered statistically significant.

## 3 Results

### 3.1 Child health staff reports on video feedback for primary screening

#### 3.1.1 Questionnaire results

A questionnaire survey was administered among child health staff participating in the program. A total of 58 questionnaires were returned; 7 were deemed invalid due to missing key information or inconsistent responses. Ultimately, 51 valid questionnaires were included in the analysis. Among the respondents, 1 was male and 50 were female; 28 were aged ≤39 years and 23 were aged ≥40 years. Eight had a junior college degree and 43 had a bachelor’s degree. Twenty-one respondents were nurses and 30 were physicians. Nineteen held junior-level titles or below, and 32 held intermediate-level titles or above. Years of practice were ≤10 years for 19 respondents, 11–20 years for 17 respondents, and >20 years for 15 respondents. Duration of work in child health was ≤5 years for 16 respondents, 6–10 years for 19 respondents, and ≥11 years for 16 respondents (**Table 1**).

**Table 1:** Demographic characteristics of participants.

Demographic variable	N(%)
Gender	
Male	1(1.96)
Female	50(98.04)
Age	
≤39	28(54.90)
≥40	23(45.10)
Education Level	
Associate	8(15.69)
Bachelor	43(84.31)
Doctor/Nurse	
Doctor	30(58.82)
Nurse	21(41.18)
Job Title	
Junior and Below	19(37.25)
Intermediate and Above	32(62.75)
Duration of Employment	
≤10 Years	19(37.25)
11–20 Years	17(33.33)
>20 Years	15(29.41)
Duration of Work in Children’s Health Care	
≤5 Years	16(31.37)
6–10 Years	19(37.25)
≥11 Years	16(31.37)

Among the survey respondents, 7 reported serving as team leaders, 36 as team members, and 8 as part-time team members. Forty respondents reported spending 0–59 minutes per week on video recording, whereas 11 reported spending ≥1 hour. Regarding the average number of recordings per video, 17 reported recording once, 32 reported recording 2–4 times, and 2 reported recording ≥5 times. Thirty-six respondents rated the quality of their team’s videos as good and 15 as average. Forty-five respondents rated the quality of other teams’ videos as good and 6 as average. All respondents reported that video-feedback learning was helpful for their work. Forty-eight respondents considered video-feedback learning to be more efficient than traditional approaches; only 3 considered its efficiency comparable to or lower than that of traditional approaches. Forty-six respondents reported that, after video-feedback learning, they would be more likely to perform well in similar situations encountered in subsequent work (**Table 2**).

**Table 2:** Relevant behaviors and opinions of respondents in video feedback learning.

Behavior/Opinion Category	N(%)
Team Roles	
Leader	7(13.73)
Team Member	36(70.59)
Part-time Member	8(15.69)
Weekly Time Allocated for Video Filming	
0-59min	40(78.43)
≥1hr	11(21.57)
Average Number of Filming Sessions per Video	
1	17(33.33)
2-4	32(62.75)
≥5	2(3.92)
Self-assessment of Team Filming Quality	
Good	36(70.59)
Average	15(29.41)
Evaluation of Other Teams’ Filming Quality	
Good	45(88.24)
Average	6(11.76)
Whether video feedback is helpful for learning	
Helpful	51(100)
Unhelpful	0(0)
Efficiency of Video Feedback Method Compared to Traditional Learning Models	
Higher	48(94.12)
At the Same Level or Lower	3(5.88)
In the screening process, when encountering similar situations in videos, are you able to complete the work effectively?	
Be able to complete the work effectively	46(90.20)
Not be able to complete the work effectively	5(9.80)

Regarding the perceived advantages of the video-feedback approach compared with traditional methods, 47 respondents reported that video feedback allows repeated viewing to identify and correct procedural details; 45 reported that videos can be viewed at any time, providing flexible learning schedules; and 45 reported that joint viewing facilitates collaborative learning and discussion, enabling participants to recognize strengths and weaknesses in their own and others’ practices. In addition, 17 respondents indicated that they felt more relaxed during video recording and that the recordings more closely reflected routine practice, and 26 respondents reported that video feedback helped provide educational guidance to parents regarding ASD. Conversely, 5 respondents stated that they preferred traditional learning methods (**Table 3**).

**Table 3:** Advantages of the video feedback method compared to traditional methods as perceived by participants.

Advantage/Preference Category	N(%)
Advantages of the Video Feedback Method Compared to Traditional Methods	
Can be watched repeatedly to identify and correct detailed issues	47(92.16)
Can be viewed at any time, allowing for flexible learning schedules	45(88.24)
Can be watched collaboratively, fostering idea exchange and identifying strengths and weaknesses in one’s own and others’ operations	45(88.24)
Recording videos helps to create a more relaxed state, closer to everyday working conditions	17(33.33)
Contributes to parents’ understanding and educational awareness of ASD	26(50.98)
Prefer traditional learning methods	5(9.80)

#### 3.1.2 Interview summary

The interviews were conducted by two project physicians from the district maternal and child health institution, both of whom had experience in qualitative research methods. Five subthemes were identified: perceptions of the training, implementation of video feedback, perceived support for learning, safety concerns, and experiences with supervision using video feedback. Participants expressed generally consistent views regarding the video-feedback approach, and thematic saturation was reached. Overall, community child health staff reported good acceptability of video-feedback discussions. Participants commonly perceived video feedback as an efficient approach for consolidating screening skills and as important for the rapid implementation of primary-level screening in community settings. With respect to training perceptions, many participants indicated that video-feedback learning facilitated mastery of key screening points, such as eye contact and the child’s social-communication status.

Participants also indicated that video feedback not only enabled community screening teams to identify potential problems during video recording, but also supported more intuitive peer learning during cross-community video exchange by allowing teams to observe and emulate strengths in other teams’ practices and to identify and correct common issues. The video format was also perceived as conducive to repeated viewing and learning. In addition, some participants noted that each community was required to submit only one to two videos for presentation, which was not perceived as adding substantial workload; the process of video submission was also considered motivating for improving screening quality in order to produce higher-quality videos.

Regarding safety concerns related to video recording, when asked whether parents might refuse or be uncooperative due to concerns about exposure of personal information, participants reported that parental informed consent was obtained prior to video recording and that parental cooperation was sought accordingly. When discussing the acceptability of using video feedback for technical supervision in primary care, participants indicated that smartphone-based video recording was convenient. Recording in routine practice environments was perceived as promoting a more natural screening state and as better reflecting real-world workflow.

### 3.2 BOT performance in primary screening

Across the 13 community health service centers, 23 teams submitted two rounds of videos. Paired t-test results indicated that (**Table 4**), following video-feedback learning, team performance improved in several checklist items, including examiner position during screening (*P* = 0.022), management of parental interference (*P* = 0.029), standardized verbal instructions (*P* = 0.002), and use of physical gestures during language items (*P* = 0.016), with statistically significant pre–post differences. In addition, the total video score increased after video-feedback learning, and the difference was statistically significant (*P* < 0.001).

**Table 4:** Primary screening physicians’ proactive observation (two behavior observations) outcomes.

Observation Indicator	1^st^ Time (Mean ± SD)	2^nd^ Time (Mean ± SD)	Difference (Mean ± SD)	*P*
Reasonable Position	0.78 ± 0.42	1	-0.22 ± 0.42	0.022
Equipment	0.87 ± 0.34	1	-0.13 ± 0.34	0.083
Screening Environment	0.91 ± 0.29	0.87 ± 0.34	0.04 ± 0.47	0.665
Parental Interference	0.52 ± 0.51	0.96 ± 0.21	-0.35 ± 0.71	0.029
Guidance Language	0.52 ± 0.51	0.96 ± 0.21	-0.43 ± 0.59	0.002
Initiation Timing	0.65 ± 0.49	0.87 ± 0.34	-0.22 ± 0.67	0.135
Waiting Time	0.48 ± 0.51	0.74 ± 0.45	-0.26 ± 0.75	0.110
Number of Operations	0.91 ± 0.29	1	-0.09 ± 0.29	0.162
Physical Actions in Language Items	0.65 ± 0.49	0.96 ± 0.21	-0.30 ± 0.56	0.016
Sequence of Operations	0.87 ± 0.34	1	-0.13 ± 0.34	0.083
Total Score	7.17 ± 1.40	9.26 ± 0.45	-2.09 ± 1.56	<0.001

The difference was calculated as the first assessment minus the second assessment.

### 3.3 Changes in the primary screening screen-positive rate during the video-reporting period

The program was initiated in June 2021. The first round of project supervision was conducted in July 2021, and video-feedback activities for community child health staff were implemented from August to October 2021. Over the course of implementation, the primary screening screen-positive rate increased progressively: 0.86% in June, 1.26% in July, 1.52% in August, 2.19% in September, and 2.43% in October, eventually approaching a relatively stable level. This change in the screen-positive rate is reported to describe screening implementation during program rollout and should not be interpreted as direct evidence of improved screening accuracy.

## 4 Discussion

Previous studies have suggested that delays in ASD diagnosis are primarily attributable to low screening uptake, suboptimal performance of screening tools in routine practice, and failure of screen-positive children to receive timely follow-up assessment. Accordingly, when the ASD screening–intervention program was implemented in Jiading District, program requirements emphasized that >80% of staff across the 13 community health service centers should effectively master the key operational points of primary-level screening, thereby ensuring that children within the catchment area could participate in screening and assessment in a timely and appropriate manner. Within the Shanghai Child Mental Health Program, primary ASD screening for children aged 18–24 months was specified to use the CHAT-A questionnaire. The present project adopted a two-step screening framework combining a parent questionnaire and the BOT; any indication of high risk in either component was classified as a positive primary screening result, with the intention of minimizing missed cases. The emphasis of the present study was on evaluating the degree of standardization in primary-level operational procedures and the role of video feedback in enhancing mastery of key operational points.

Physician-led behavioral observation refers to structured, as-natural-as-possible observation of a child’s responses during routine well-child visits at 18–24 months of age, and constitutes a foundation for objective and effective primary screening. Training for professionals involved in developmental and behavioral screening typically requires substantial time and strict standards; before reaching a level of independent advanced practice, personnel generally need individual practice, self-assessment, and repeated behavioral observation. When primary screening is implemented broadly in community settings as a full-coverage program requirement, frontline staff must achieve comprehensive competency in screening procedures. One month after completion of program training, supervision conducted by three groups across the 13 community child health outpatient clinics indicated that only one community met the required quality standard for primary screening, whereas the remaining clinics required retraining (5).

### 4.1 Video feedback is acceptable to frontline staff in terms of workload and delivery format

When initiating retraining, it is necessary to consider that frontline clinical work is often high-volume and complex. Successful implementation depends on acceptance and engagement by frontline staff; if training content or workload is substantially misaligned with real-world conditions, clinicians may be more likely to disengage or participate passively (6). Under the video-feedback approach, frontline staff did not need to leave their workplaces; approximately two hours per week were devoted to team-based discussion and completion of a video recording, and one representative per month spent approximately 15 minutes receiving face-to-face feedback. This format was relatively low in workload, convenient, and generally acceptable to participating staff (7).

During video feedback, members who were unable to attend in-person discussions due to work schedules, external rotations, or meetings could benefit indirectly from the video interpretation provided by attending colleagues. Under conventional on-site guidance, frontline staff may experience persistent pressure or challenge. In particular, discussing procedural details in front of parents during on-site feedback may increase parental anxiety; meanwhile, onsite staff may withhold issues or avoid discussion due to fear of making mistakes. In contrast, within video-feedback sessions, community teams could present strengths, weaknesses, and errors and explicitly identify them. This process of self-identifying shortcomings may further promote active reflection on how to improve primary ASD screening skills.

### 4.2 Sharing and evidence-based reflection within video feedback facilitate capacity-building among frontline staff

During on-site supervision, both supervisors and frontline staff observe the assessment process from their own perspectives, and blind spots may remain. By contrast, video feedback can replay the workflow and allow attention to each operational step. Video sharing, within the structured framework provided by video feedback, can promote self-observation under relatively natural conditions (8). Staff have the opportunity to watch their own performance from a third-person perspective (9) and to develop insights into structuring operational procedures across assessments. Interview data indicated that many community child health teams collaborated in preparing videos; when a selected video was perceived as low quality, teams engaged in self-review and re-recorded the procedure in anticipation of presenting higher-quality assessment videos during feedback sessions. Across three rounds of shared review, the use of a unified framework enabled staff from the 13 community centers to share goals and achievements in improving primary ASD screening skills (10), articulate reflections on procedural improvement, and engage in collegial, video-based analysis and sharing. This process may support a shift from minimal, episodic learning toward a continuous development process grounded in shared thinking and methods (11). The development of collegial relationships in routine training may also represent a strategy contributing to training success and to high-quality attainment of subsequent program objectives (12).

### 4.3 Reinforced presentation of detail through video feedback enhances frontline staff’s perception and learning

Across the three training sessions, differences were observed in the time required for improvement across the eight quality-control elements. Environmental and equipment-related factors are relatively amenable to standardization (13) and therefore may be easier for frontline staff to align. However, certain nonverbal or subtle communication-related elements—such as the waiting interval between the first and second name-calling attempts when assessing response to name, or inadvertent body language during procedures—were more difficult to recognize and master. During on-site supervision, each element is typically presented within similar time constraints; in video feedback, however, these less conscious elements can be replayed repeatedly (14), drawing attention to them and guiding staff to use video feedback as a self-observation tool. With practice, teams can revise and improve these elements in subsequent video recordings.

### 4.4 Enhancing frontline physicians’ behavioral observation skills may broaden the scope of developmental–behavioral work in primary care

#### 4.4.1 Supporting parental education for children at high risk for ASD

During primary care well-child visits, when a child is identified as high risk, frontline physicians are required to guide parents and facilitate adequate understanding of heterogeneity among high-risk children. Some parents may believe that the child is merely developing more slowly and will gradually “catch up,” which can delay further clinical evaluation and undermine the value of early screening and early intervention.

As frontline physicians’ behavioral observation skills improve—particularly with better mastery of initiation timing, waiting time, number of operations, use of physical gestures during language items, and operational sequence—more nuanced understanding may develop regarding behaviors such as reduced speech, reduced eye contact, reduced responsiveness, and reduced pointing. In addition, discrepancies between physician-led observation and parent-initiated prompts may help clinicians identify parental misunderstandings (15). As a result, clinicians may be better positioned to communicate core interpretations of high-risk behaviors in a timely manner, potentially increasing the likelihood that parents proceed to further diagnostic evaluation.

#### 4.4.2 Supporting families of children diagnosed with ASD in primary care

Post-diagnostic intervention for ASD is long-term and often requires substantial family time and financial investment (14). Social support is therefore essential. ASD intervention is comprehensive; in addition to behavioral intervention, nutrition, growth and development, and behavioral assessment are also important. If community physicians master behavioral observation skills and integrate them with routine child health monitoring competencies, and subsequently provide supportive health education, they may serve as a meaningful source of social support (16), thereby strengthening families’ capacity to sustain intervention efforts.

#### 4.4.3 Supporting flexible approaches to screening in primary care

Research on AI-based technologies for ASD screening is expanding; however, nationwide coverage may require additional time. Because AI equipment is typically site-fixed, implementation in more remote areas may remain challenging in the near term. If frontline child health physicians possess behavioral observation skills, children may be observed and assessed across multiple settings and time points, enhancing flexibility of primary-level assessment.

In broadly implemented primary-level skills training, no single method can meet all educational needs. The present study indicates that video feedback—through recording routine practice and sharing feedback aligned with procedural steps—can provide a relatively accessible and convenient means of supporting team-based improvement in procedural quality among frontline staff (17).

## Data Availability

The raw data supporting the conclusions of this article will be made available by the authors, without undue reservation.
